# Novel B cell epitopes mapping in pD205R protein of African swine fever virus using monoclonal antibodies

**DOI:** 10.1186/s12917-026-05559-9

**Published:** 2026-05-18

**Authors:** Chen Lang, Yilu Zhang, Yingying Zhang, Wenqin Gong, Danyang Zhang, Zixiang Zhu, Juan Bai, Ping Jiang, Yanni Gao

**Affiliations:** 1 Key Laboratory of Animal Diseases Diagnostic and Immunology, Ministry of Agriculture, MOE Joint International Research Laboratory of Animal Health and Food Safety, College of Veterinary Medicine, Nanjing Agricultural University, Nanjing, 210095 China; 2 State Key Laboratory of Animal Disease Control and Prevention, College of Veterinary Medicine, Lanzhou University, Lanzhou Veterinary Research Institute, Chinese Academy of Agricultural Sciences, Lanzhou, 730046 China; 3 Jiangsu Co-Innovation Center for the Prevention and Control of Important Animal Infectious Diseases and Zoonoses, Yangzhou University, Yangzhou, 225009 China

**Keywords:** African swine fever virus, PD205R protein, Monoclonal antibodies, B cell epitopes

## Abstract

**Background:**

African swine fever virus (ASFV) is a highly significant pathogen that causes hemorrhagic fever in domestic pigs and wild boars, resulting in substantial economic losses to the swine industry worldwide. The D205R protein (pD205R), which exhibits high similarity to the eukaryotic subunit 5 of RNA polymerase II (RPB5), has been identified as a component of the ASFV virion. However, the biological functions of pD205R remain largely unexplored.

**Results:**

In this study, the recombinant pD205R (rpD205R) protein was expressed using a prokaryotic expression system, and five monoclonal antibodies (mAbs) targeting pD205R were generated. Among them, four mAbs − 5C8, 5C10, 6D5 and 9H8 − were identified as belonging to the IgG2a isotype with a kappa light chain, while mAb 3A5 was of the IgG1 isotype with a kappa light chain. The specific recognition of rpD205R by all five mAbs was demonstrated by Western blot and indirect immunofluorescence assay (IFA). Moreover, mAbs 3A5, 5C8, 5C10 and 6D5 exhibited strong reactivity with the native viral pD205R protein. Furthermore, two novel linear epitopes, spanning amino acid residues 144QEEAQEFLGR153 and 174LGGRPGDFVQ183, were identified by epitope mapping using these mAbs. Sequence alignment analysis revealed that both epitopes are conserved among the analyzed representative strains.

**Conclusions:**

In this study, five monoclonal antibodies—designated 3A5, 5C8, 5C10, 6D5, and 9H8—targeting the ASFV pD205R protein were successfully generated, resulting in the identification of two novel linear epitopes. These findings not only offer valuable monoclonal antibody tools for future investigations into the biological functions of D205R but also contribute to the development of ASFV serological diagnostic methods.

**Supplementary Information:**

The online version contains supplementary material available at 10.1186/s12917-026-05559-9.

## Background

African swine fever (ASF) is a viral hemorrhagic disease affecting domestic pigs and wild boars, caused by the African swine fever virus (ASFV). The disease is characterized by an acute infection with a mortality rate that can approach 100% [1]. The current global ASF pandemic began in 2007 following the introduction of the virus from East Africa into the Caucasus region. It subsequently spread into Eastern Europe and Russia, and has more recently emerged in Western Europe and Asia [2]. While ASFV infection often presents with few clinical signs in wild boars, it causes severe symptoms in domestic pigs, including hemorrhages in internal organs, mucoid diarrhea, and skin reddening [3, 4]. In the absence of internationally recognized safe and effective vaccines, ASF has inflicted substantial economic losses on the global swine industry.

ASFV is the sole member of the Asfarviridae family. It is a large, enveloped virus with a linear double-stranded DNA genome of 170 − 194 kbp, encoding more than 150 polypeptides [5]. The viral proteins are involved in critical processes such as virion assembly, regulation of virus replication, DNA replication and repair, gene expression, and evasion of host defenses. Among these, proteins such as p30, p54, p72, p49, p11.5, p14.5, p22, p34, pI329L, pK205R and CD2v have been primarily selected as serological targets for ASFV detection [6–13]. Conversely, proteins including pEP153R, pA137R, pE120R, pMGF505 − 7R, pI267L, pI177L and pF317L have been implicated in virus − host interactions and are being explored for vaccine development[14–19]. Despite these advances, the biological functions of approximately half of the ASFV-encoded proteins remain uncharacterized.

The D205R protein (pD205R) is one such protein of interest. The carboxy − terminal half of pD205R shares high sequence similarity with eukaryotic subunit 5 of Pol II (RPB5), and contains the signature domain of this protein superfamily (RNA − pol − Rpb5 − C). This structural homology suggests a potential role for pD205R in viral transcription and mRNA modification [20–22]. Its presence in highly purified extracellular ASFV particles, as identified by mass spectroscopy, further supports a function in early viral transcription [23]. Moreover, high − abundance expression of pD205R has been detected from 12 h post-infection (hpi) in both porcine alveolar macrophages (PAMs) and MA104 cells, indicating its potential as an antigenic target for ASFV detection [24]. Collectively, these findings suggest that pD205R may be a multifunctional protein during ASFV infection. Therefore, investigating its biological roles could provide novel insights into viral pathogenesis and identify potential targets for ASFV prevention and control strategies.

In the present study, we generated and characterized five monoclonal antibodies (mAbs) against ASFV pD205R and subsequently identified two novel linear B cell epitopes. The antigenic characteristics of pD205R, combined with the sequence conservation analysis of these epitopes, suggest that pD205R may serve as a valuable target for ASF surveillance. This work contributes to the functional characterization of ASFV pD205R and provides foundational tools for the advancement of ASFV immunodiagnostics.

## Results

### Preparation of recombinant pD205R protein

**Fig. 1:**
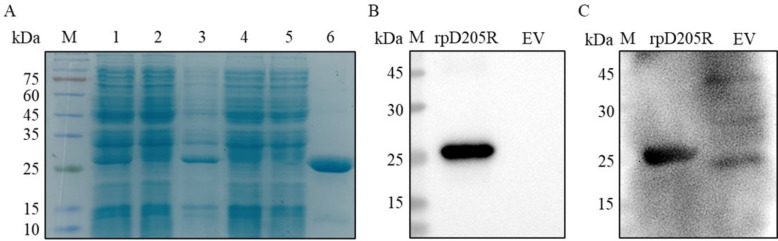
Analysis of rpD205R protein expression and purification. **A** SDS − PAGE analysis of His − tagged rpD205R protein expression followed by Coomassie brilliant blue staining. Lane M, protein ladder; lane 1, whole − cell lysates of IPTG − induced bacteria (pColdI-D205R); lane 2, supernatant fraction of IPTG − induced bacterial lysates (pColdI-D205R); lane 3, pellet fraction of IPTG − induced bacterial lysates (pColdI-D205R); lane 4, whole − cell lysates of uninduced bacteria (pColdI-D205R); lane 5, whole − cell lysates of IPTG − induced bacteria (pColdI vector without insert); lane 6, purified rpD205R protein. **B** Western blot analysis of rpD205R protein using an anti − His tag mAb. **C** Western blot analysis of rpD205R protein using anti − ASFV positive serum

The recombinant plasmid pColdI-D205R was constructed and subsequently transformed into *Escherichia coli* (*E.coli*) Rosetta (DE3) to express rpD205R. As illustrated in Fig. 1A, the rpD205R (26.5 kDa) was expressed predominantly in the form of inclusion bodies in the lysates of *E.coli* cells following IPTG induction. Following denaturation using urea and subsequent renaturation through gradient dialysis, the rpD205R was purified via affinity chromatography using HisSep nickel − nitrilotriacetic acid (Ni − NTA) agarose resin column. The purified protein demonstrated strong immunoreactivity, as it was specifically recognized by both anti − His mAb and anti − ASFV positive serum, respectively (Fig. 1B and C).

### Development of anti − pD205R mAbs

**Fig. 2:**
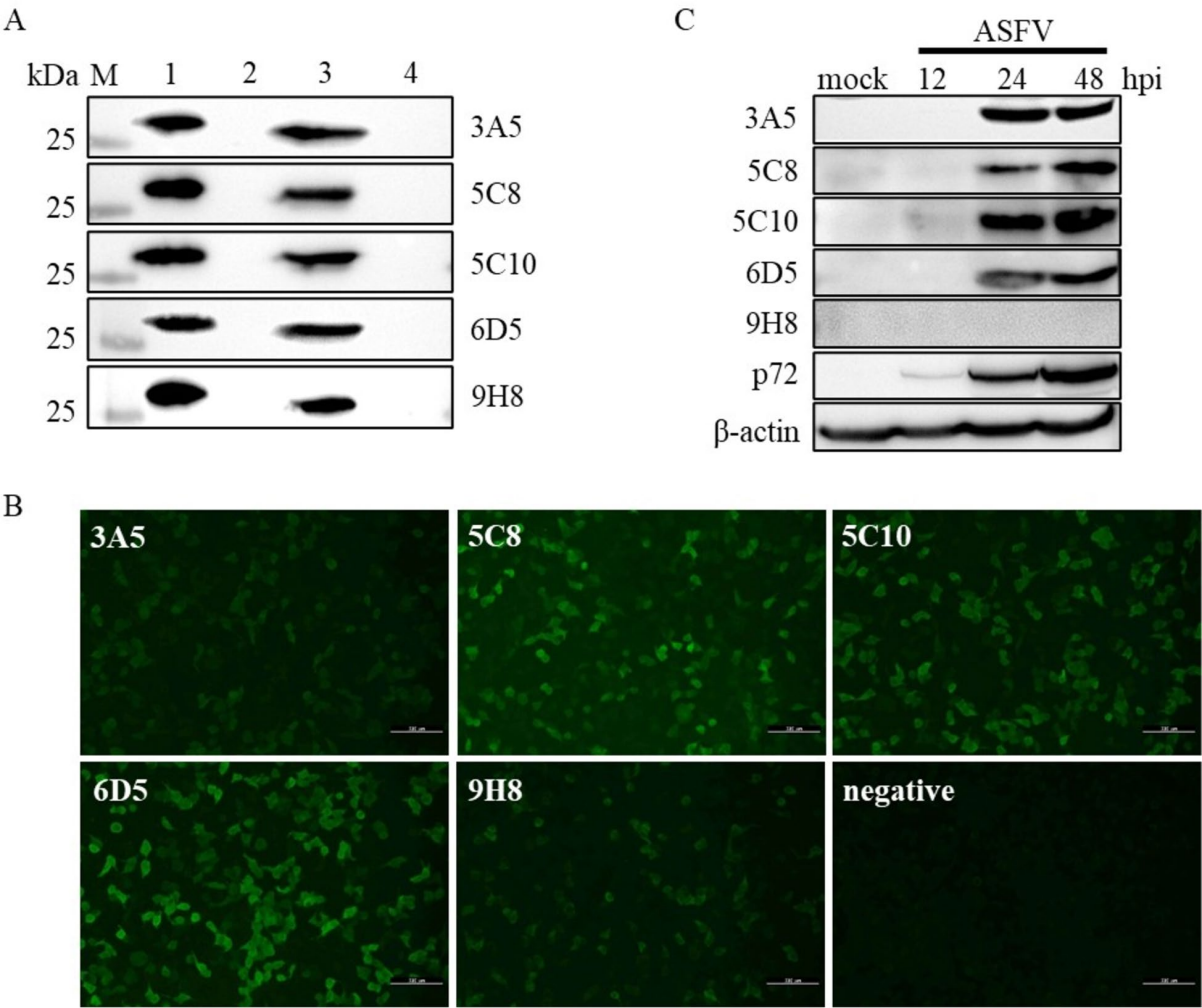
Reactivity of the anti − rpD205R mAbs. **A** Western blot analysis of anti − rpD205R mAbs with *E.coli* − expressed rpD205R protein and HEK − 293 T − expressed rpD205R protein. Lane 1, purified *E.coli* − expressed rpD205R protein; lane 2, *E.coli* cell lysates containing empty pColdI vector (negative control); lane 3, lysates of HEK − 293 T cells transfected with pCAGGS − D205R; lane 4, lysates of HEK − 293 T cells transfected with empty pCAGGS vector (negative control). **B** IFA analysis of anti − rpD205R mAbs recognizing HEK − 293 T − expressed rpD205R protein. HEK − 293 T cells transfected with pCAGGS-D205R were incubated with the indicated rpD205R − specific mAbs (annotated in the top left of each panel), followed by staining with Coralite488 − conjugated AffiniPure Goat Anti − Mouse IgG(H + L). Scale bars, 20 μm. **C** Western blot analysis of anti − rpD205R mAbs reactivity with viral pD205R expressed in ASFV-infected Marc-145 cells. Cell lysates were prepared from Marc-145 cells infected with ASFV (MOI = 10) and harvested at the indicated time points post-infection (12, 24, and 48 hpi). Mock-infected cell lysates served as a negative control

Five hybridoma cell lines, designated 3A5, 5C8, 5C10, 6D5 and 9H8, were generated against rpD205R. The specificity of these five mAbs was assessed by Western blot analysis using both prokaryotically expressed rpD205R and eukaryotically expressed rpD205R (24.7 kDa), employing the respective mAbs as primary antibodies. All five mAbs demonstrated robust reactivity and high specificity towards both forms of rpD205R (Fig. 2A). Indirect immunofluorescence assay (IFA) performed on HEK − 293 T cells transfected with the pCAGGS − D205R construct revealed specific green fluorescence signals (Fig. 2B). As Marc-145 cells has been shown to support productive infection of the ASFV strain (CN/GS/2018) used in this study [25], ASFV − infected Marc-145 cells was used to evaluate the capacity of the prepared mAbs to recognize the native viral proteins. The results indicated that mAbs 3A5, 5C8, 5C10 and 6D5 exhibited strong reactivity with the viral pD205R, while 9H8 failed to recognize the viral antigen (Fig. 2C).

### Isotype determination of the mAbs

The isotypes of the mAbs were determined with the supernatant of hybridoma cells using a Mouse Monoclonal Antibody Isotyping Elisa Kit. Among them, 3A5 belonged to IgG1 subclass and harbored the κ light chain, whereas 5C8, 5C10, 6D5 and 9H8 were identified as IgG2a antibodies, possessing the κ light chain (Table 1).

**Table 1:** Monoclonal antibodies isotype identification

Monoclonal Antibody	Subclass and type							
	**IgG1**	**IgG2a**	**IgG2b**	**IgG3**	**IgA**	**IgM**	**κ**	**λ**
3A5	**0.455**	0.058	0.059	0.058	0.058	0.087	**0.169**	0.059
5C8	0.059	**1.093**	0.058	0.058	0.057	0.053	**0.232**	0.058
5C10	0.059	**1.052**	0.059	0.059	0.062	0.062	**0.295**	0.061
6D5	0.058	**1.013**	0.057	0.06	0.061	0.058	**0.17**	0.059
9H8	0.058	**1.183**	0.057	0.06	0.058	0.059	**0.281**	0.058

Positive results were marked in bold

### Identification of rpD205R epitopes

**Fig. 3:**
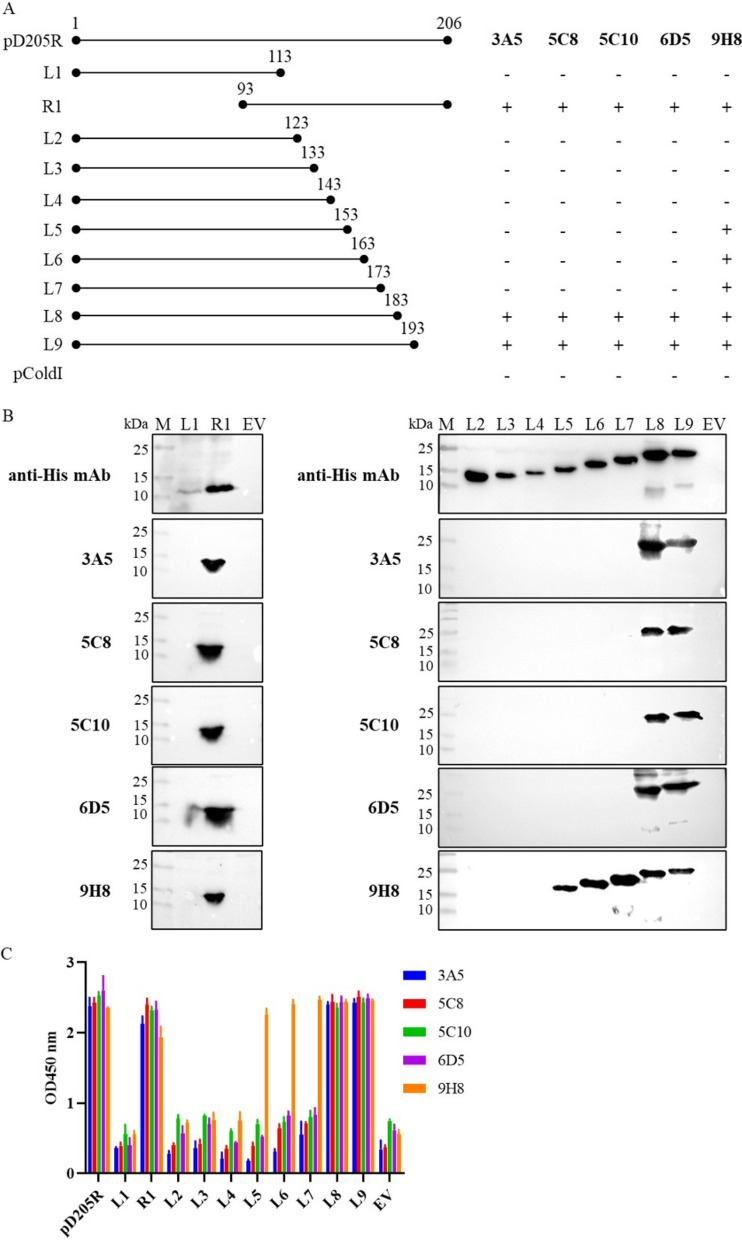
Epitope identification of anti-pD205R mAbs. **A** Schematic representation of the strategy used to map the epitopes within the pD205R protein and summary of reactivity between the truncated rpD205R protein fragments and the five mAbs. The full length pD205R and a series of truncated fragments (L1-L9 and R1) were constructed as His-tagged fusion proteins. Reactivity was determined by Western blot analysis and is indicated as follows: +, positive interaction; −, no interaction. **B** Western blot analysis confirming the reactivity of the five mAbs with the truncated pD205R fragments. Lysates of *E.coli* expressing the various His-tagged truncated pD205R fragments (L1-L9 and R1) were probed with anti-His mAb and each of the five mAbs (3A5, 5C8, 5C10, 6D5, and 9H8). **C** Peptide-based ELISA analysis validating the reactivity of the five mAbs with the truncated pD205R fragments

To delineate the epitopes recognized by the five prepared mAbs, ten overlapping peptides spanning the pD205R were employed in Western blot analysis (Fig. 3A). Reactivity patterns of the mAbs with fragments L1 and R1 localized the antibody − binding region to amino acids 114–206 of pD205R. Subsequent fine mapping using truncated fragments L2 − L9 revealed that mAbs 3A5, 5C8, 5C10 and 6D5 recognized a common epitope corresponding to residues 174LGGRPGDFVQ183, while mAb 9H8 specifically recognized a distinct epitope at 144QEEAQEFLGR153 of pD205R (Fig. 3B). Peptide-based ELISA was performed to validate the Western blot results (Fig. 3C).

### Characteristics analysis of the epitopes

**Fig. 4:**
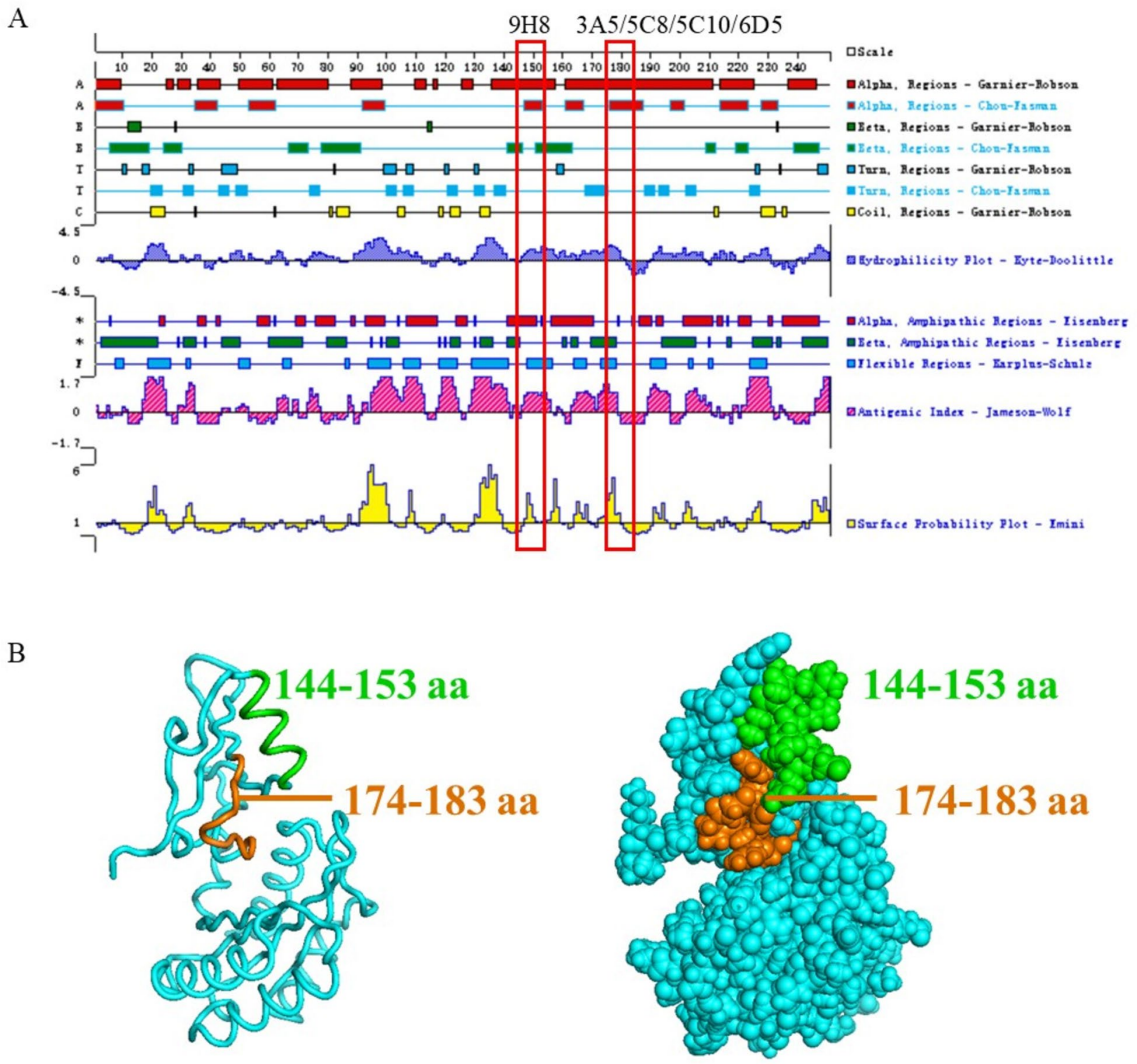
Characterization of identified B cell epitopes within the pD205R protein. **A** Bioinformatic analysis of the antigenic properties of the identified epitopes. The antigenic index, surface accessibility, and flexible regions of pD205R were predicted using the DNAStar Protean 7.0. The positions of the two minimal epitopes were indicated by boxes. **B** Three-dimensional structural localization of the identified epitopes on the pD205R protein rendered using PyMOL software

The characteristics of the two identified epitopes were predicted using DNAstar Protean 7.0 software. Epitope 144QEEAQEFLGR153 was predicted to exhibit an alpha helix and a beta sheet structure, whereas epitope 174LGGRPGDFVQ183 was predicted to adopt an alpha‑helical conformation. Neither of these two epitopes contains turn or coil structures; instead, they are predominantly surface‑exposed and display high hydrophilicity and antigenicity (Fig. 4A). Structural analysis using PyMOL corroborated these predictions, revealing that 144QEEAQEFLGR153 was located within a protruding bulge structure on the protein surface, whereas 174LGGRPGDFVQ183 was partially surface-accessible (Fig. 4B).

### Conservation analysis of epitopes

**Fig. 5:**
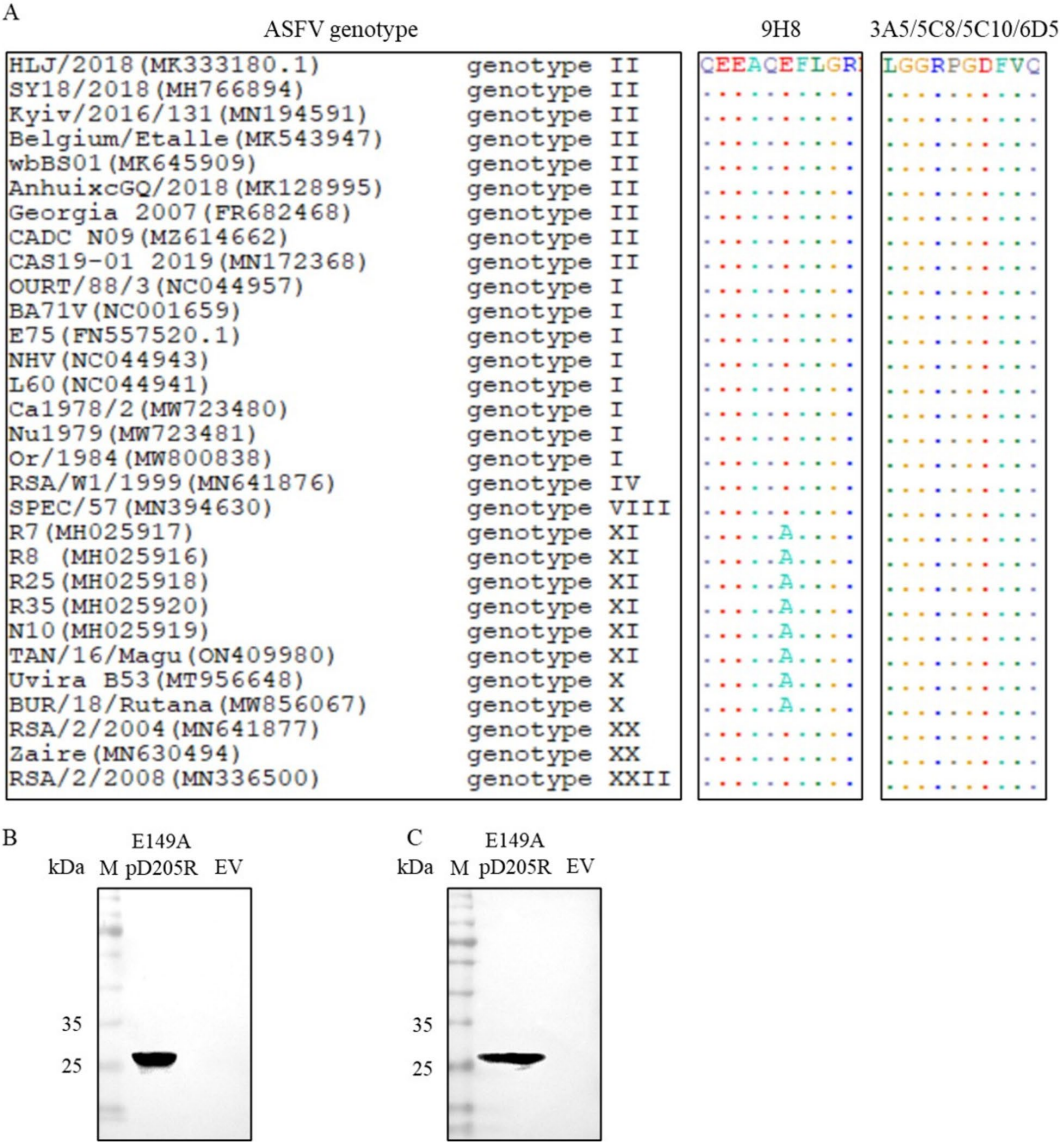
Conservation analysis of epitopes. **A** Homology analysis of the identified epitope region. The amino acid sequences of pD205R from 30 representative ASFV strains available in GenBank were aligned and compared. **B** Identification of the E149A − rpD205R mutant by Western blot using an anti − His tag antibody. **C** Reactivity of the 9H8 mAb with the E149A − rpD205R mutant

Conservation analysis of the identified epitopes was conducted using Jalview Version 2 with 30 ASFV strains obtained from GenBank, among which eight strains were representative of genotype I, nine strains represented genotype II, and 13 strains corresponded to other genotypes. As illustrated in Fig. 5A, the epitope 174LGGRPGDFVQ183 was found to be conserved among all ASFV genotypes examined. In contrast, the epitope 144QEEAQEFLGR153 was conserved among genotypes I, II, IV, VIII, XX and XXII, but exhibited a glutamic acid-to-alanine substitution at position 149 (E149A) in genotypes X and XI. To further investigate whether the 9H8 mAb could recognize the corresponding epitope in ASFV genotypes X and XI, His-tagged E149A − rpD205R was expressed and verified with anti − His tag mAb (Fig. 5B). Further Western blot analysis demonstrated that 9H8 mAb could also recognize E149A − rpD205R, indicating that the amino acid substitution present in genotypes X and XI did not abrogate the reactivity between the 9H8 mAb and the pD205R protein (Fig. 5C).

## Discussion

ASF is a severe and highly contagious disease affecting domestic pigs and wild boars, notifiable to the World Organization for Animal Health, and poses a substantial threat to the global swine industry and food security. The development of safe and effective antiviral strategies necessitates the identification of immunogenic viral antigens, as well as a comprehensive understanding of the pathogenic mechanisms and immune evasion strategies employed by ASFV. However, the large number of viral proteins produced during ASFV replication renders the comprehensive and precise identification of diagnostic and vaccine targets. Therefore, it is imperative to elucidate the biological characteristics of individual viral proteins to provide novel insights for the design of antiviral interventions. The ASFV pD205R was initially identified in purified ASFV particles [23] and exhibits high sequence similarity to the eukaryotic RPB5 [20]. Subsequent studies have shown that pD205R is abundantly expressed during ASFV infection and plays an integral role in the viral replication process [24]. Collectively, these findings suggested that pD205R might represent a promising target for ASFV diagnosis and vaccine development. Nevertheless, the precise biological function and antigenic properties of pD205R remain to be fully elucidated.

In the present study, the rpD205R protein was expressed in a prokaryotic system using *E.coli*. and its immunoreactivity was validated with both anti − His tag mAb and anti-ASFV positive serum. The strong reactivity observed between rpD205R and the anti-ASFV positive serum indicated favorable antigenicity of the recombinant protein, suggesting that pD205R protein may serve as a viable target for ASFV diagnostic applications. Subsequently, using hybridoma technology, five mAbs-5C8, 5C10, 6D5, 9H8 (all of the IgG2a/κ isotype), and 3A5 (IgG1/κ isotype)-were generated. The specificity of these five mAbs were confirmed by Western blot and IFA employing rpD205R proteins expressed in both prokaryotic and eukaryotic systems. However, when evaluating the reactivity of these mAbs against the native viral pD205R protein in ASFV − infected Marc-145 cells by Western blot, it was unexpectedly observed that mAb 9H8 failed to recognize the viral protein. It is hypothesized that, during viral infection, the critical amino acid residues recognized by mAb 9H8 may undergo post-translational modifications that are resistant to removal under denaturing conditions, thereby abrogating their reactivity with this specific mAb.

The mapping of epitopes within functional viral protein is of considerable significance, as these epitopes represent potential targets for both vaccine design and the development of antiviral therapeutics. In this study, two novel linear B cell epitopes, localized within 144QEEAQEFLGR153 and 174LGGRPGDFVQ183, were identified using the five generated mAbs. The epitope 144QEEAQEFLGR153, recognized by mAb 9H8, was predicted to be hydrophilic, highly antigenic and surface-exposed, suggesting it may constitute an active site susceptible to post − translational modification, which is consistent with our aforementioned conjecture. Sequence conservation analysis of the two identified epitopes, performed across 30 ASFV strains representing eight distinct genotypes (I, II, IV, VIII, XI, X, XX and XXII), demonstrated that the 174LGGRPGDFVQ183 epitope was absolutely conserved among all ASFV strains examined, suggesting that mAbs 3A5, 5C8, 5C10 and 6D5 possess broad − spectrum reactivity and are proposed to be suitable for application in the surveillance of diverse ASFV genotypes. In contrast, a single amino acid substitution (E149A) within the 44QEEAQEFLGR153 was identified in ASFV genotype X and XI. Notably, this mutation did not impair the reactivity between mAb 9H8 and the E149A − rpD205R mutant protein, indicating that glutamic acid at position 149 is not a critical residue for 9H8 recognition. Given that mAb 9H8 does not recognize the native viral pD205R protein, its utility may be primarily confined to studies investigating the functional properties of the pD205R protein. Another two epitopes of pD205R protein have previously been revealed. One is 96VLSKKNI102, fully exposed on the protein surface and showing highly conservation within ASFV strains and elevated antigenicity and hydrophilicity [24]. The other previously identified epitope, 167SDPPVVWLGGRPGD180, was reported to have critical amino acids including S167, W173, L174, G175, P178, and D180 [26]. Although this epitope partially overlaps with our newly identified epitope (174LGGRPGDFVQ183), the distinct key residues suggest that they are different epitopes.

Cross-reactivities of the five prepared mAbs against porcine RPB5 were also investigated. Firstly, the amino acid sequences of the porcine RPB5 protein and the ASFV pD205R protein, with particular focus on the conservation of the epitope-containing sequences, were analyzed. The results revealed that RPB5 and pD205R share five identical amino acids (E145, E146, E149, L151, R153) within epitope 144QEEAQEFLGR153, and three identical amino acids (G175, G179, V182) within epitope 174LGGRPGDFVQ183. Subsequently, we evaluated the reactivity of the prepared five mAbs against porcine cells (PK-15 and ST cells) by Western blot. No specific reactivity with porcine cells was observed for 5C8, 5C10, 6D5, and 9H8, whereas 3A5 produced nonspecific bands with both cell types. However, the molecular weights of the specific bands were significantly larger than that of the porcine RPB5 protein (24.6 kDa) (Fig. S1). Therefore, it is speculated that the five mAbs showed no cross-reactivities with porcine RPB5 protein.

Nonetheless, several limitations of the present study should be acknowledged. First, although the prokaryotic expression system in *E.coli* is the most commonly used platform for producing recombinant proteins, using proteins expressed in this system as immunogens to generate mAbs still presents certain limitations. These include: the lack of post-translational modifications in prokaryotic proteins, which precludes the generation of antibodies that recognize modified epitopes; or improper protein folding, leading to the absence or misformation of conformational epitopes. Moreover, only linear B cell epitopes were explored in this study, which may not adequately capture the complete antigenic repertoire of the protein. More investigations should be carried out to identify conformational epitopes which could also contribute to the antigenicity and functions of pD205R protein. In addition, for development of diagnostic methods for ASFV, the reaction specificity (i.e., the absence of cross-reactivity with other porcine viruses) and stability of the antibodies prepared in this study should be validated. Furthermore, to serve as a reference for antiviral strategies, the key amino acids within the epitopes need to be further elucidated, and the antigenicity of the identified epitopes in swine immune system also requires further investigation.

## Conclusion

In summary, five monoclonal antibodies − 3A5, 5C8, 5C10, 6D5, and 9H8 − targeting the ASFV pD205R protein were successfully generated in this study, leading to the identification of two novel epitopes. These findings not only provide valuable tools for functional studies of D205R but also contribute to the advancement of ASFV serological diagnosis.

## Materials and methods

### Cells, virus, serum and animals

SP2/0, Marc-145 and HEK − 293 T cells were preserved in our laboratory. Mouse myeloma SP2/0 cells were cultured in Roswell Park Memorial Institute (RPMI) − 1640 medium (Gibco, Waltham, MA, USA) supplemented with 10% heat − inactivated fetal bovine serum (FBS; Lonsera, Canelones, Uruguay), penicillin (250 U/mL), streptomycin (250 µg/mL), and 1% L − glutamine. Marc-145 and HEK − 293 T cells were maintained in Dulbecco’s modified Eagle’s medium (DMEM; Gibco, Waltham, MA, USA) containing 10% FBS (Lonsera, Canelones, Uruguay), penicillin (250 U/mL), and streptomycin (250 µg/mL). The ASFV genotype II strain CN/GS/2018, originally isolated using PAMs at the Lanzhou Veterinary Research Institute, was employed in this study. Anti − ASFV positive and negative reference sera were procured from Pudao (Beijing) Standard Technology Co., Ltd. Female BALB/c, aged five to six weeks and specific pathogen − free, were obtained from Yangzhou University Experimental Animal Center.

### Construction of the recombinant expression vector

Primers for the amplification of the D205R gene (Table 2) were designed based on the corresponding gene sequence of ASFV Pig/HLJ/2018 strain (GeneBank accession no. MK333180.1). For the construction of pColdI − D205R, the D205R gene was amplified using the primers pColdI − D205R − PF and pColdI − D205R − PR and subsequently cloned into the pColdI vector utilizing the *Xho*I and *EcoR*I restriction sites via T4 DNA ligase (New England Biolabs, Beverly, MA, USA). The resulting recombinant plasmid was transformed into *E.coli* DH5α competent cells (Vazyme, Nanjing, China) before incubation on kanamycin − containing agar plates at 37 ℃ for 12 h. The recombinant plasmid was verified by DNA sequencing (Sangon Biotech Co., Ltd., Shanghai, China). For the construction of pCAGGS − D205R recombinant plasmid, the D205R gene was amplified with primers pCAGGS − D205R − PF and pCAGGS − D205R − PR and cloned into the pCAGGS vector using *EcoR*I and *Xho*I restriction sites. The recombinant plasmid was transformed into DH5α competent cells, subjected to selection with ampicillin, and confirmed as described above.

**Table 2:** Primers for amplification of fragments spanning D205R gene

Fragments	Primers	Sequences (5’ − 3’)	Position
pColdI − D205R	PF	CGCTCGAGATGGCCATGCAAAAGTTATTTAC	1 − 206 aa
	PR	CGGAATTCTCAAATTTTGGACTTGGTG	
pCAGGS − D205R	PF	CGCGAATTCATGGCCATGCAAAAGTTA	1 − 206 aa
	PR	CGCCTCGAGTCAAATTTTGGACTTGGTG	
L1	PF	CGGAATTCATGGCCATGCAAAAGTTATTTAC	1 − 113 aa
	PR	CGCTCGAGTCAATTTGCAGCGCGCTGCTC	
R1	PF	CGGAATTCATGAAGCCCGTTTTAAGCAAAA	93 − 206 aa
	PR	CGCTCGAGTCAAATTTTGGACTTGGTG	
L2	PF	CGCTCGAGATGGCCATGCAAAAGTTATTTAC	1 − 123 aa
	PR	CGGAATTCTCAATCAACGATTAAAATTAATTCTTC	
L3	PF	CGCTCGAGATGGCCATGCAAAAGTTATTTAC	1 − 133 aa
	PR	CGGAATTCTCATAAAATATTTTTTTTGCTTAAAAC	
L4	PF	CGCTCGAGATGGCCATGCAAAAGTTATTTAC	1 − 143 aa
	PR	CGGAATTCTCAGTAGGGATATATGTTTATTAC	
L5	PF	CGCTCGAGATGGCCATGCAAAAGTTATTTAC	1 − 153 aa
	PR	CGGAATTCTCACACCTTGGGAATGTTAATG	
L6	PF	CGCTCGAGATGGCCATGCAAAAGTTATTTAC	1 − 163 aa
	PR	CGGAATTCTCAAGTAATTAGTTTATGTTTAG	
L7	PF	CGCTCGAGATGGCCATGCAAAAGTTATTTAC	1 − 173 aa
	PR	CGGAATTCTCAGCGACCTAAAAACTCCTG	
L8	PF	CGCTCGAGATGGCCATGCAAAAGTTATTTAC	1 − 183 aa
	PR	CGGAATTCTCATTGCATGAGGTCCTGC	
L9	PF	CGCTCGAGATGGCCATGCAAAAGTTATTTAC	1 − 193 aa
	PR	CGGAATTCTCACCAGACCACCGGGGGG	
pColdI − D205R − E149A	PF-1	CGCTCGAGATGGCCATGCAAAAGTTA	1 − 457 bp
	PR-1	CGACCTAAAAACGCCTGCGCCTCC	
	PF-2	GGAGGCGCAGGCGTTTTTAGGTCG	437 − 618 bp
	PR-2	CGGAATTCTCAAATTTTGGACTTGGTGATA	

### Induced expression, purification and identification of recombinant pD205R (rpD205R) protein

The recombinant 6 × His − tagged rpD205R was expressed in *E.coli* Rosetta (DE3) (Vazyme, Nanjing, China) transformed with pColdI − D205R recombinant plasmid. Bacterial cells were cultured in Luria − Bertani (LB) medium at 37 ℃ until the optical density at 600 nm (OD600) reached 0.5 − 0.6. Expression of rpD205R was induced by the addition of 1 mM IPTG, followed by incubation at 16 ℃ for 8 h. Cell lysates were subsequently analyzed by sodium dodecyl sulfate − polyacrylamide gel electrophoresis (SDS − PAGE). For rpD205R protein purification, the cell cultures were centrifuged at 4000 × g for 20 min before the precipitation was resuspended in cold phosphate buffered saline (PBS) for sonication on ice (300 W, cycles of 5 s pulses followed by 5 s intervals, for a total of 20 min). The rpD205R protein, which was present in the insoluble fraction of the cell lysates, was denatured using urea, renatured through gradual dialysis, and subsequently purified by Ni − NTA affinity chromatography. The purified rpD205R was analyzed and confirmed by SDS − PAGE and Western blot using an anti − His mAb (Proteintech Group, Inc.,Rosemont, IL, USA). The immunoreactivity of the purified rpD205R was further verified by Western blot with anti − ASFV positive serum.

### Preparation of anti − rpD205R mAbs

Monoclonal antibodies against rpD205R were generated using hybridoma technology as previously described [27]. Briefly, the purified rpD205R was emulsified with an equal volume of incomplete Freund’s adjuvant ((Sigma − Aldrich (Shanghai) Trading Co., Ltd., Shanghai, China) to prepare the immunogen. Five female BALB/c mice (six weeks old) were immunized subcutaneously with 80 µg of the prepared antigen per mouse, for a total of three immunizations administered at three-week intervals. After three rounds of immunization, the antiserum titer was evaluated by means of an indirect enzyme-linked immunosorbent assay (iELISA). Animals whose serum displayed iELISA titers above 1:409,600 following the third immunization were selected. Seven days following a booster immunization, splenocytes harvested from the immunized mice were fused with SP2/0 cells using PEG4000 (Sigma − Aldrich (Shanghai) Trading Co. Ltd., Shanghai, China). The fused cells were cultured in HAT selection medium for seven days, after which the culture supernatants were screened for the presence of anti-rpD205R antibodies by iELISA in three repeats. The determination of positive wells relied on iELISA data, where a well was considered positive when the ratio of its optical density (OD) reading to that of the negative control reached or exceeded 2.1. Positive hybridoma cells were subsequently subcloned by limiting dilution to establish monoclonal cell lines stably producing anti-rpD205R mAbs.

### Coomassie blue staining and Western blot analysis

The prokaryotically expressed rpD205R was prepared using the method described above. For eukaryotic expression, rpD205R was produced by transfecting the pCAGGS − D205R plasmid into HEK − 293 T cells, and cell samples were collected at 24 h post transfection (hpt). To evaluate the reactivity of the anti − pD205R mAbs with ASFV, Marc-145 cells seeded in 12-well plates were infected with ASFV at a multiplicity of (MOI) of 10 and collected at 12, 24 and 48 hpi. Protein samples were mixed with loading buffer, heated at 100 ℃ for 10 min, and subsequently subjected to SDS-PAGE for separation of denatured proteins. For Coomassie blue staining, the gels were stained with staining solution for 30 min and then destained with destaining solution prior to visualization. For Western blot analysis, proteins separated by SDS − PAGE were transferred onto nitrocellulose membranes and blocked with 5% skimmed milk at room temperature for 4 h. After five washes with PBST, the membranes were incubated with primary antibodies (anti-His mAb, anti-ASFV positive serum, or the prepared anti-pD205R mAbs) for 2 h at room temperature. Following another five washes, the membranes were incubated with HRP − labelled goat anti-mouse or anti-pig IgG secondary antibodies for 45 min incubation at room temperature. After a final wash, the immunoreactive bands were visualized and analyzed using a digital imaging system. Each experiment was independently repeated two to three times, and images shown in this study are representative of multiple experiments.

### IFA

To assess the reactivity of the prepared mAbs with the eukaryotically expressed rpD205R protein, HEK − 293 T cells were transfected with pCAGGS-D205R plasmid. At 48 hpt, cells were fixed with 4% paraformaldehyde (PFA) for 30 min at room temperature, followed by permeabilization with 0.2% Triton X-100 in PBS for 10 min at room temperature. Subsequently, the cells were blocked with 2% bovine serum albumin (BSA) in PBS for 3 h at room temperature. The cells were then incubated with the primary for 2 h at room temperature, followed by incubation with Coralite488 − conjugated AffiniPure Goat Anti − Mouse IgG(H + L) (Proteintech Group, Inc., Rosemont, IL, USA) as the secondary antibody for 45 min at 37 ℃. Between each step, the cells were washed five times with PBST. Finally, fluorescence signals were visualized and analyzed using a Zeiss Axio Observer microscope (Carl Zeiss, Jena, Germany). Each experiment was independently repeated two to three times, and images shown in this study are representative of multiple experiments.

### Isotype determination of the anti-rpD205R mAbs

A Mouse Monoclonal Antibody Isotyping Elisa Kit was used to determine the isotypes of the five anti-rpD205R mAbs with the supernatant of hybridoma cells following the manufacturer's instructions.

### Epitopes mapping and analysis

To delineate the antigenic regions of rpD205R recognized by the five prepared mAbs, a peptide scanning approach was employed. Ten overlapping peptides spanning rpD205R were expressed in *E.coli* Rosetta (DE3) (primers listed in Table 2), followed by Western blot and iELISA analysis using the respective mAbs.

The characteristics of the identified B cell epitopes were predicted using DNAStar Protean 7.0 software. The spatial localizations of these epitopes on the pD205R protein structure were analyzed using PyMOL software (version 2.5.0), based on a three − dimensional model of pD205R predicted by the Robetta automated structure prediction service (https://robetta.bakerlab.org/; Accessed on 6 Dec 2025)).

Conservation analysis of the two epitopes across different ASFV genotypes was performed. Sequences of 30 viral strains, isolated at different times and areas, representing eight distinct ASFV genotypes (I, II, IV, VIII, XI, X, XX and XXII), were retrieved from the GenBank database and used for sequence alignment using Jalview (Version 2).

To investigate whether the mAb 9H8 could differentiate ASFV genotypes X and XI from other genotypes, a mutant variant of the D205R gene, designated E149A-D205R, was generated by fusion PCR using specific primers (Table 2). The corresponding mutant protein, E149A-rpD205R, was subsequently expressed and purified following the same procedure as described for the wild-type protein.

### Biosafety statement and facilities

All experiments with live ASFV in this study were performed in the biosafety level 3 lab at the Lanzhou Veterinary Research Institute.

## Supplementary Information

Supplementary Material 1.

Supplementary Material 2.

## Data Availability

All data generated or analysed during this study are included in this published article.
