# Supplementary figures and images for: Novel B cell epitopes mapping in pD205R protein of African swine fever virus using monoclonal antibodies
Source: BMC Vet Res. 2026 May 18;22:410. doi: 10.1186/s12917-026-05559-9 (PMC13352800; doi:10.1186/s12917-026-05559-9)

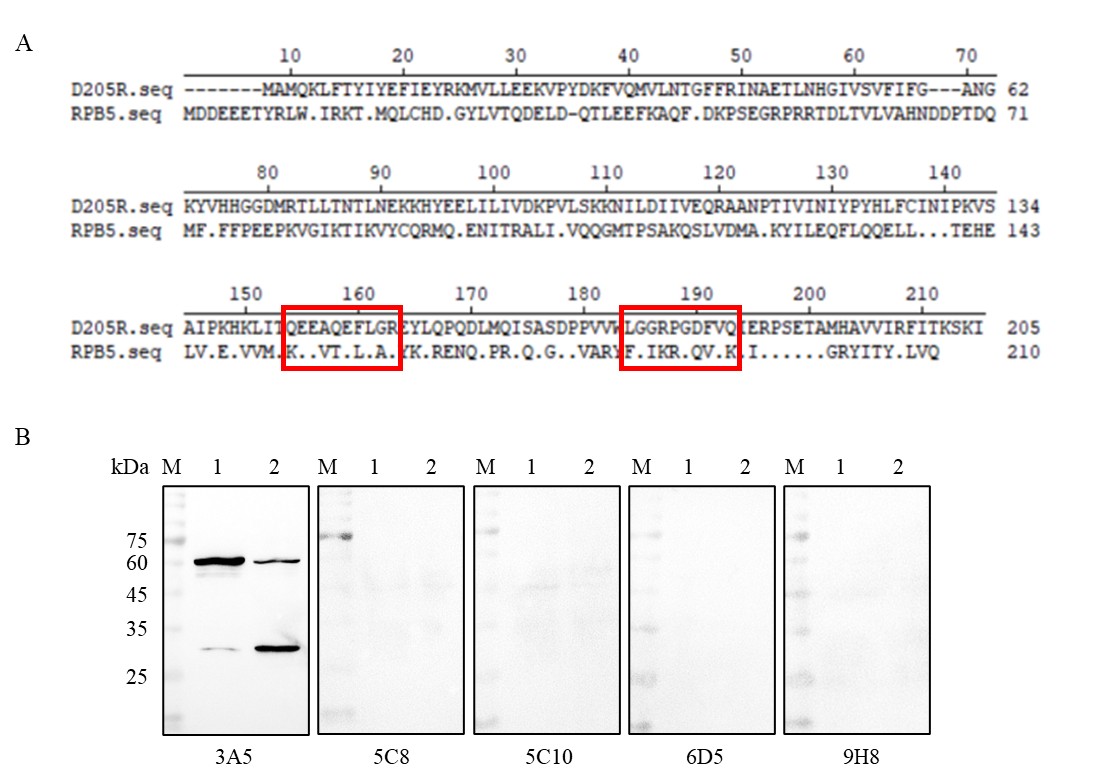

Supplement: Supplementary file 1 — Supplementary Material 1. [file 12917_2026_5559_MOESM1_ESM.jpg]
