# Supplementary material for: Novel B cell epitopes mapping in pD205R protein of African swine fever virus using monoclonal antibodies
Source: BMC Vet Res. 2026 May 18;22:410. doi: 10.1186/s12917-026-05559-9 (PMC13352800; doi:10.1186/s12917-026-05559-9)
Supplement: Supplementary file 2 — Supplementary Material 2. [file 12917_2026_5559_MOESM2_ESM.pptx]

## Slide 1
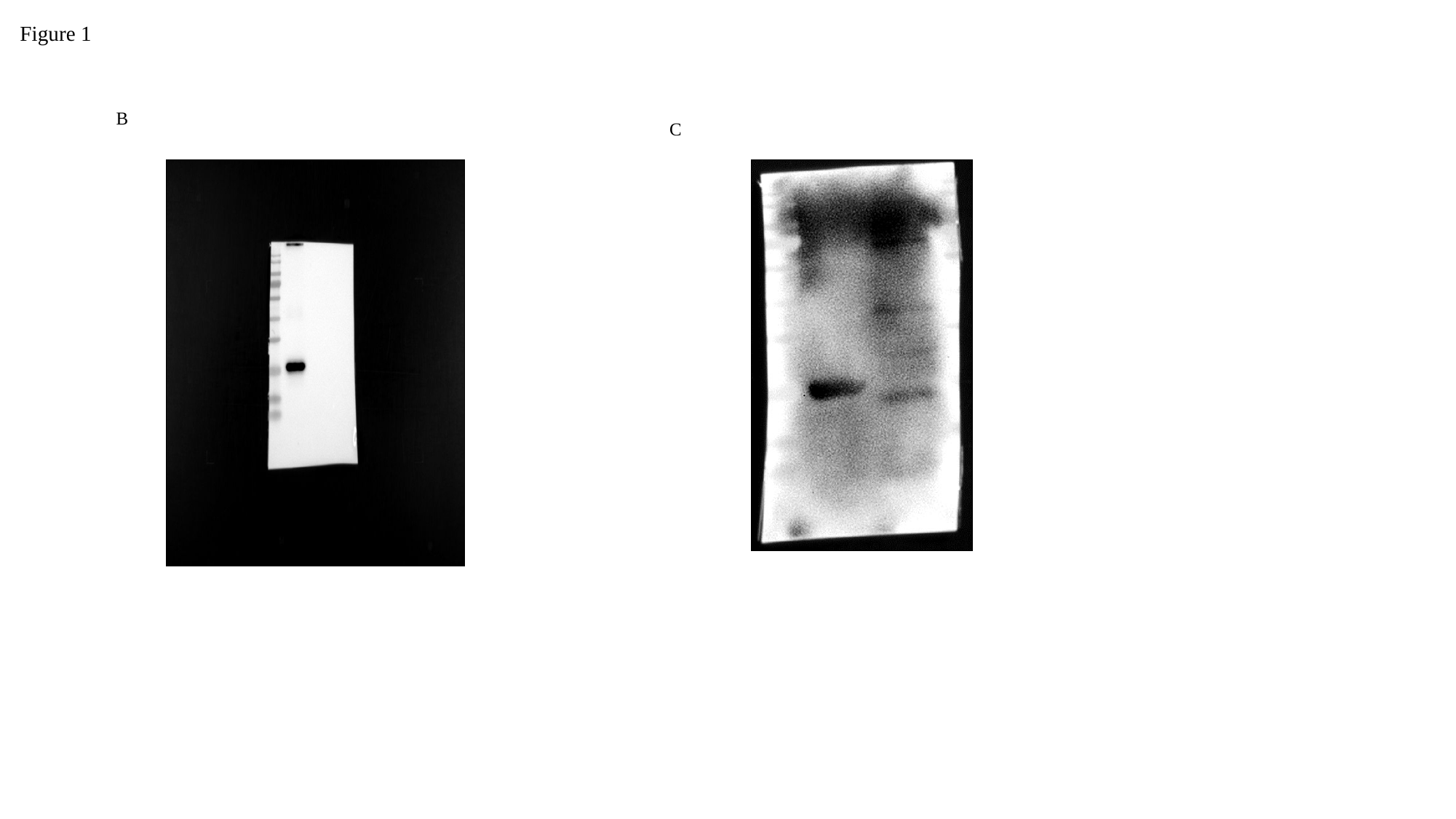

Figure 1
B
C

## Slide 2
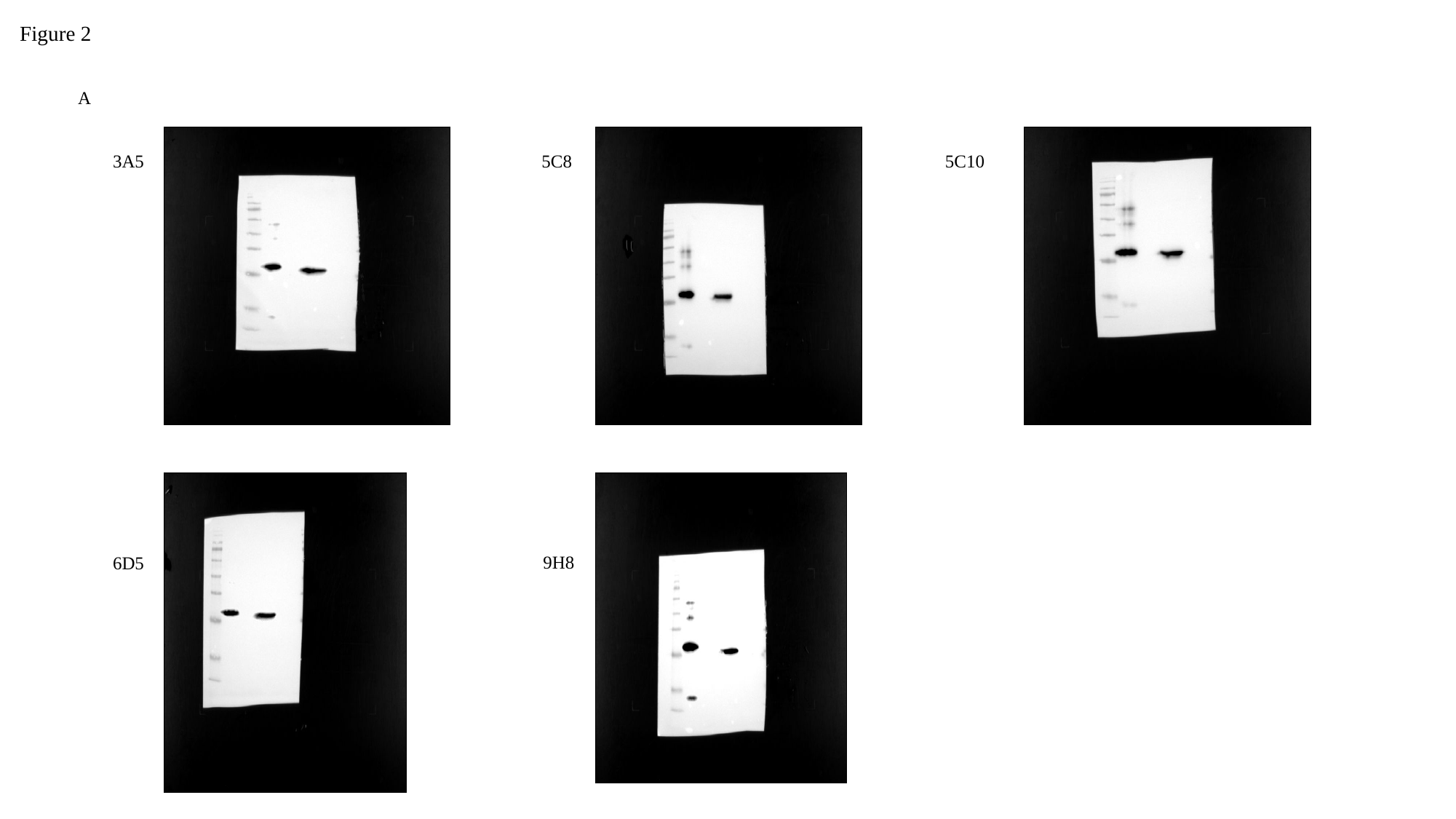

Figure 2
A
5C8
5C10
3A5
9H8
6D5

## Slide 3
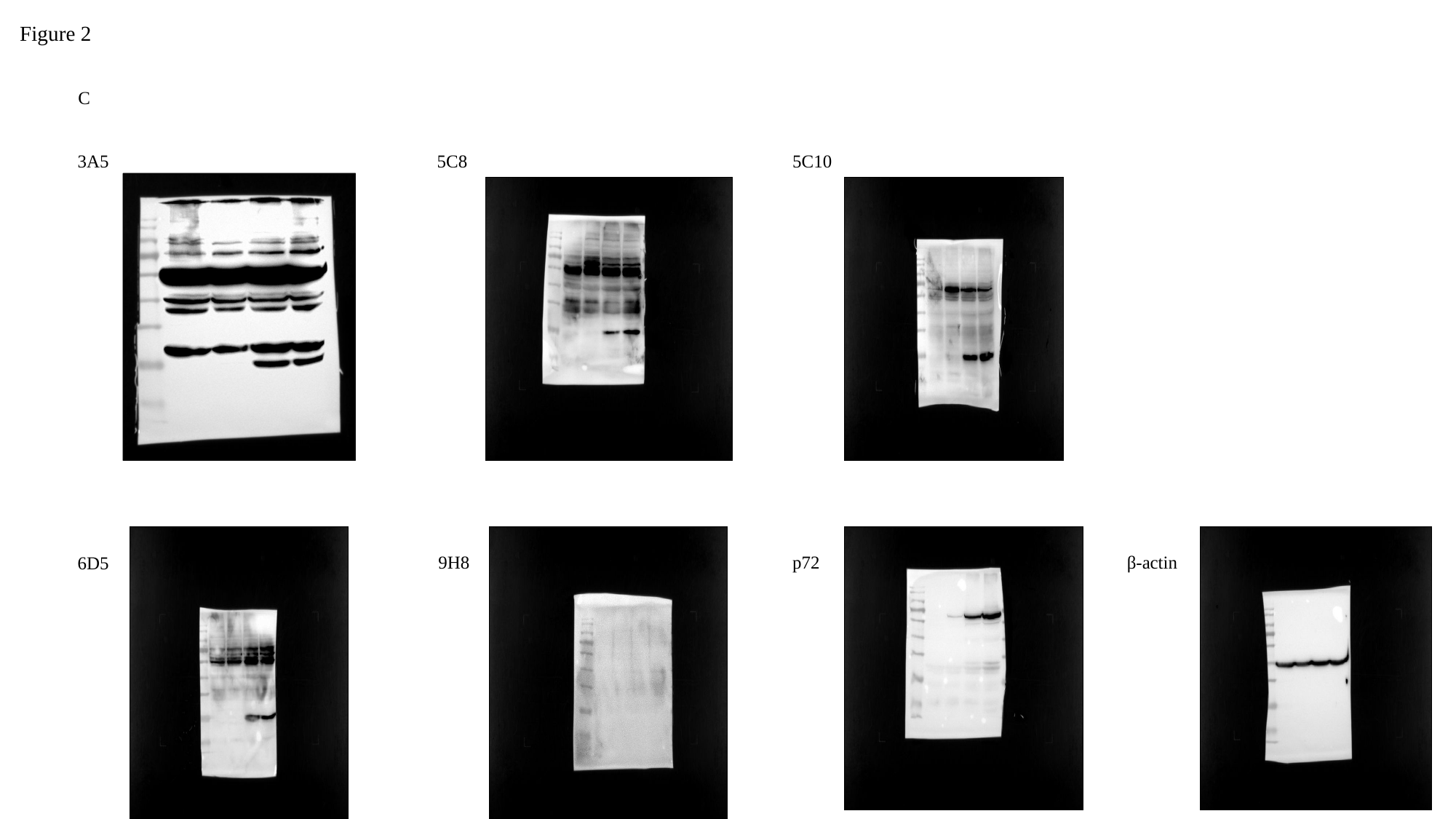

Figure 2
C
3A5
5C8
5C10
p72
β-actin
9H8
6D5

## Slide 4
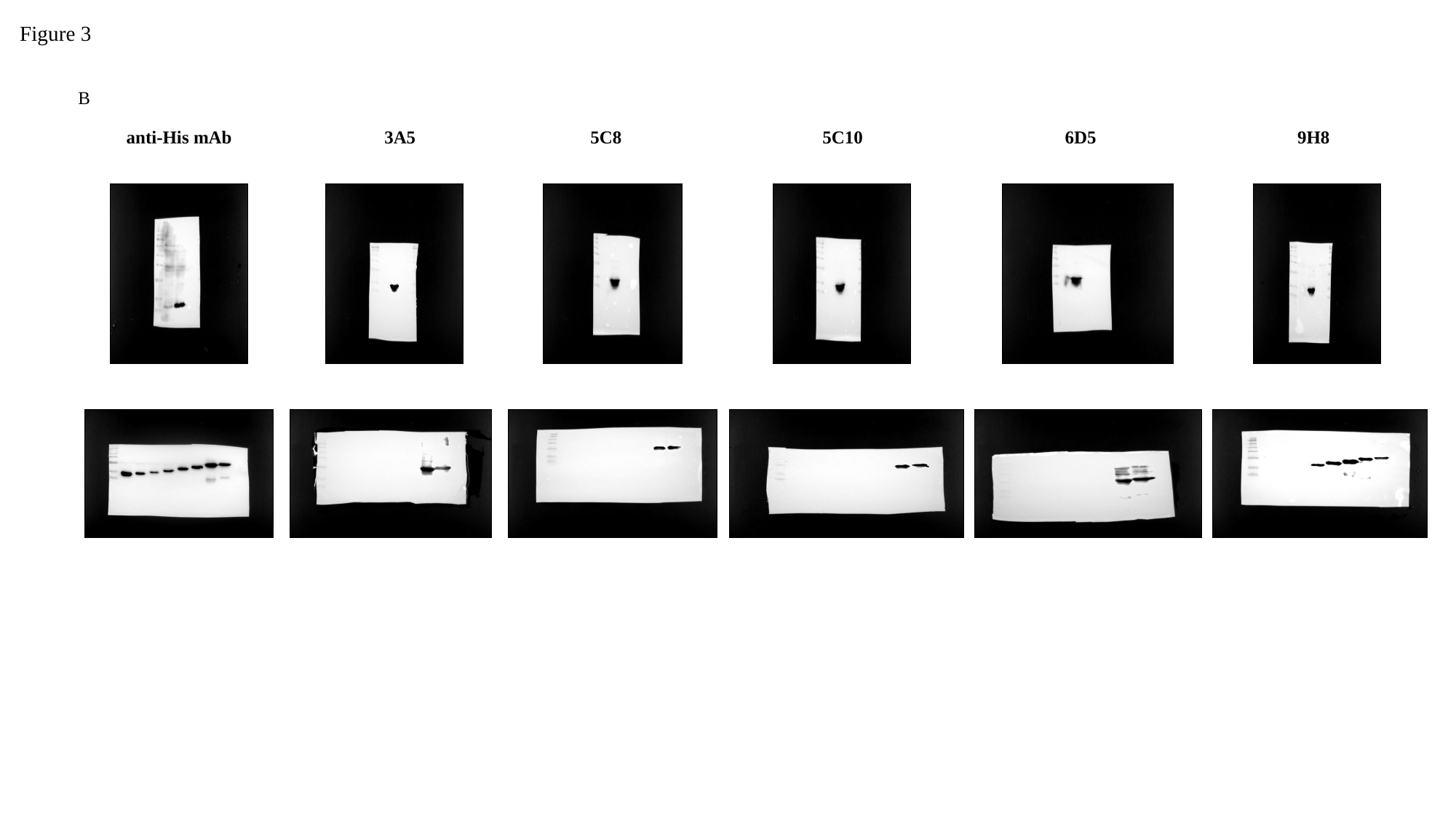

Figure 3
B
anti-His mAb
6D5
9H8
3A5
5C10
5C8

## Slide 5
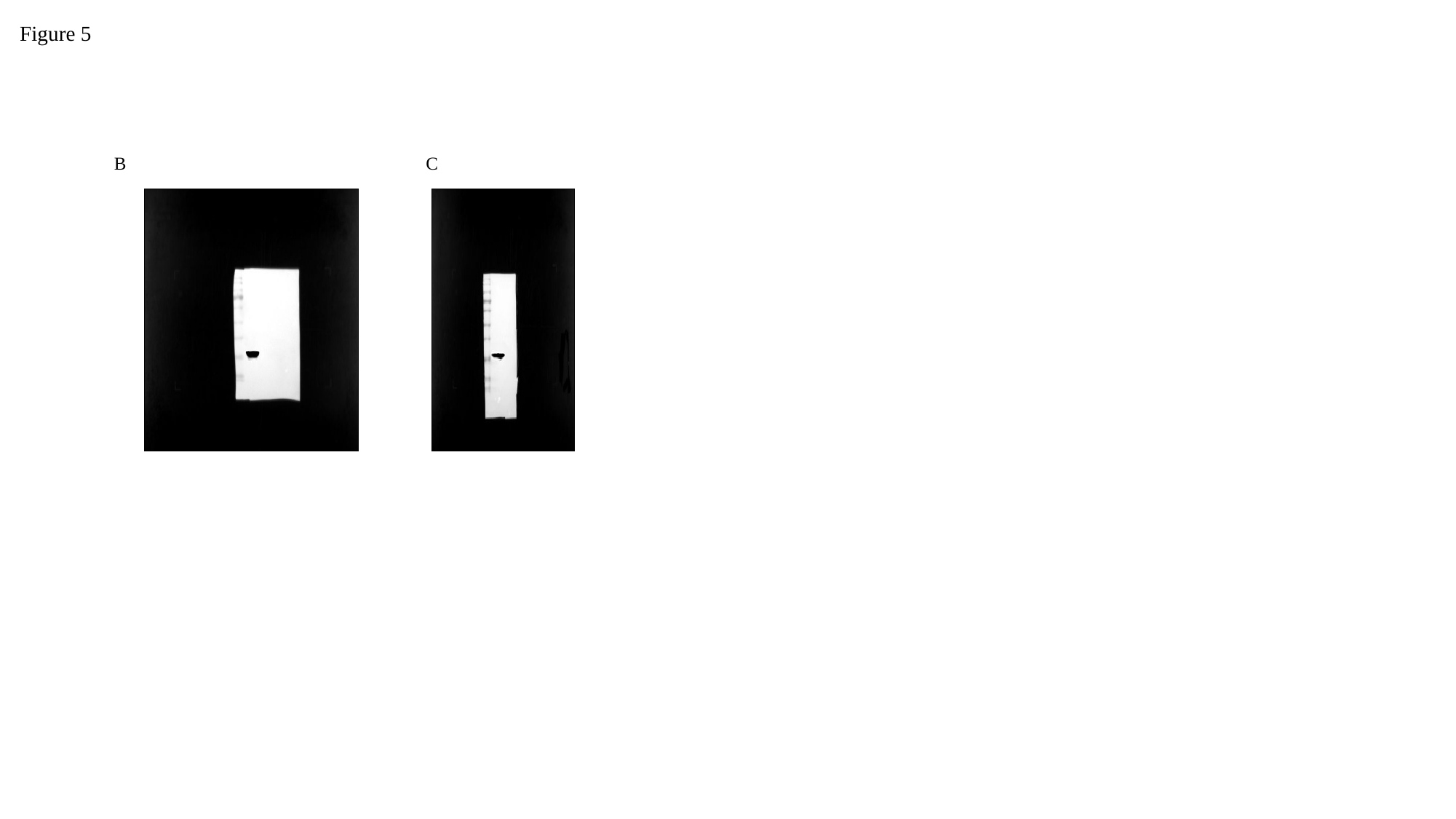

Figure 5
B
C

## Slide 6
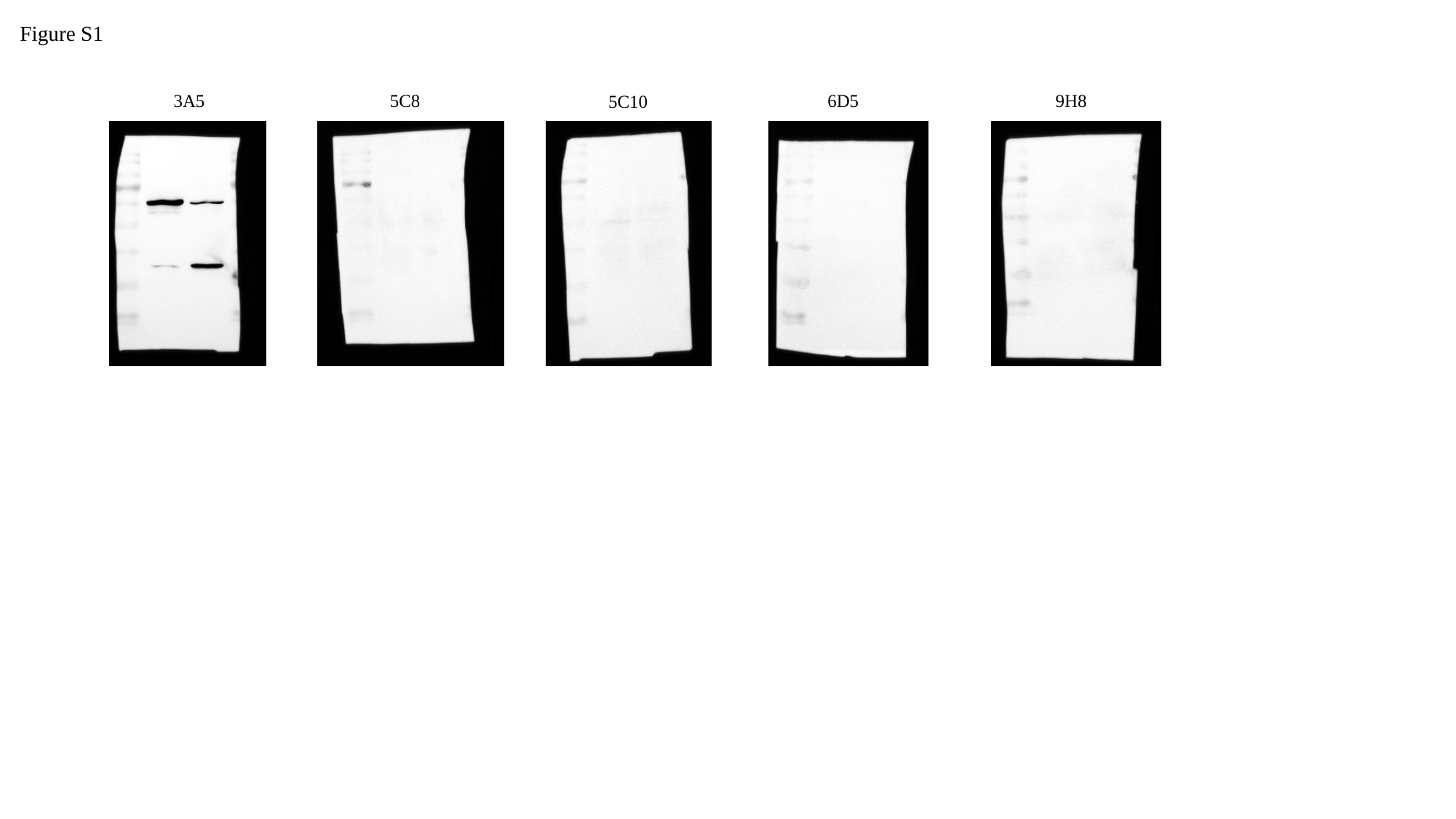

Figure S1
5C8
6D5
9H8
3A5
5C10
